# Pathogenic Network Analysis Predicts Candidate Genes for Cervical Cancer

**DOI:** 10.1155/2016/3186051

**Published:** 2016-02-29

**Authors:** Yun-Xia Zhang, Yan-Li Zhao

**Affiliations:** ^1^The 2nd Department of Gynecology, The Affiliated Tumor Hospital of Xinjiang Medical University, Urumqi, Xinjiang 830000, China; ^2^Department of Gynecology, Ninth Hospital of Xi'an, Xi'an, Shaanxi 715100, China

## Abstract

*Purpose*. The objective of our study was to predicate candidate genes in cervical cancer (CC) using a network-based strategy and to understand the pathogenic process of CC.* Methods*. A pathogenic network of CC was extracted based on known pathogenic genes (seed genes) and differentially expressed genes (DEGs) between CC and normal controls. Subsequently, cluster analysis was performed to identify the subnetworks in the pathogenic network using ClusterONE. Each gene in the pathogenic network was assigned a weight value, and then candidate genes were obtained based on the weight distribution. Eventually, pathway enrichment analysis for candidate genes was performed.* Results*. In this work, a total of 330 DEGs were identified between CC and normal controls. From the pathogenic network, 2 intensely connected clusters were extracted, and a total of 52 candidate genes were detected under the weight values greater than 0.10. Among these candidate genes,* VIM* had the highest weight value. Moreover, candidate genes* MMP1*,* CDC45*, and* CAT* were, respectively, enriched in pathway in cancer, cell cycle, and methane metabolism.* Conclusion*. Candidate pathogenic genes including* MMP1*,* CDC45*,* CAT*, and* VIM* might be involved in the pathogenesis of CC. We believe that our results can provide theoretical guidelines for future clinical application.

## 1. Introduction

Cervical cancer (CC), arising from the cervix, is a major cause of cancer death in developing countries [1]. Globally, there are approximately half a million new diagnoses and 250,000 CC-related deaths annually [2]. Metastasis is a major cause of cancer-related mortality [3]. In literature, human papillomavirus (HPV) infection is a risk factor to result in the development of CC [4]. Nevertheless, growing evidences have demonstrated that HPV infection alone is not sufficient to cause malignant initiation, and genetic alterations are essential for progression from precancerous disorder to invasive cancer [5]. Thus, it is urgent to understand the pathogenic process of CC via dissecting the components which take part in the pathogenesis, for instance, pathogenic genes, thereby preventing the development from precancerous disorder to CC.

In laboratory, several techniques, including gene silencing and knockout, were used to identify the pathogenic genes. So far, a total of 43 pathogenic genes of CC have been verified through biological experiments and deposited in Online Mendelian Inheritance in Man (OMIM) database. Nevertheless, the pathogenic genes are far from enough. Moreover, the process of identifying pathogenic genes is painful and time consuming. Fortunately, computational approaches can solve this difficulty. A large number of studies have adopted comparative genomics method to obtain differentially expressed genes (DEGs) to elucidate the pathogenic procedures of disease via comparing control and disease groups [6, 7]. However, DEGs alone may cause false positives while extracting crucial genes involved in disease procedure because some genes do not participate in the pathway of pathogenic genes even if their expression changes are significant. Moreover, studies have shown that many of gene biomarkers obtained from different researches on the same disease are typically inconsistent [8, 9]. To overcome this difficulty, a potentially more effective approach is to employ a network-based strategy to evaluate the disease-related biomarkers. For example, the integration of correlating protein interaction network and phenotype network provided by Wu and colleagues has been demonstrated to identify human disease genes with high accuracy [10]. Moreover, human genome-wide protein-protein interactions (PPIs) have been applied to detect disease-related genes by analyzing topological features in PPI network (PPIN) [11]. Importantly, Liu et al. [12] have demonstrated that integrating protein interaction map as well as gene expression data is effective to predict the pathogenic genes. Thus, the molecular interaction network of CC can give hints to potential pathogenic genes.

In an attempt to obtain novel pathogenic genes of CC, we also used the integration data of protein information and gene data. We hypothesize that interacting proteins often share parallel functions [13] and are likely involved in the similar pathways [14]. Thus, a pathogenic subnetwork composed of potential pathogenic genes is detected with a small number of known pathogenic genes as seed genes. In brief, a pathogenic network was extracted based on seed genes and DEGs. Subsequently, in order to identify the subnetworks in the pathogenic network, ClusterONE was utilized to carry out the cluster analysis. Each gene in the pathogenic network was assigned a weight value, and then candidate genes were obtained based on the weight distribution. Eventually, the pathway enrichment analysis for candidate genes was performed. The candidate genes are expected to be involved in the same biological processes as seed genes and thereby might be pathogenic genes. Our study might provide guidelines for experimental verification in the future and shed light on the pathogenesis from precancerous disorder to invasive CC.

## 2. Material and Methods

### 2.1. Data Acquisition

The microarray profile E-GEOD-39001 [15], which was under GPL201 platform of [HG-Focus] Affymetrix Human HG-Focus Target Array and GPL6244 platform of [HuGene-1_0-st] Affymetrix Human Gene 1.0 ST Array [transcript (gene) version], was downloaded from the EMBL-EBI database which is a public functional resource for gene expression data of humans. In the current study, gene microarray data of 62 HPV vaccination 16-positive CC and 17 healthy cervical epitheliums were used to perform further analysis based on these two platforms.

### 2.2. Data Preprocessing and Identification of DEGs

The original expression measures from healthy control and CC conditions were converted to expression values via robust multiarray average (RAM) [16]. The genes which were differently expressed between CC and healthy controls were identified by significance analysis of microarrays (SAM) algorithm. In brief, statistically significant genes in expression were identified using SAMR function. Each gene was distributed a score on the basis of gene expression change comparing with the standard deviation of repeated measurements for this gene. If the scores of these genes were greater than a liminal value, these were defined as potentially significant. The ratio of falsely significant genes to the significant genes was regarded as false discovery rate (FDR). To increase the stringency for significant difference, delta value was computed by means of the function of SAMR.compute.delta.table. DEGs between healthy control and CC conditions were screened out using the cut-off value of delta = 0.806.

### 2.3. Identification of Pathogenic Network

**Figure 1:**
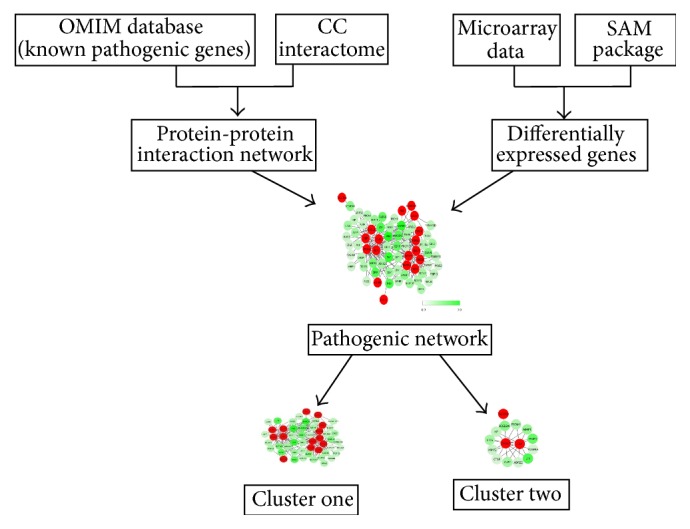
The flow diagram of a network strategy to predict pathogenic genes. First of all, the differentially expressed genes were identified. Then, a pathogenic network was extracted. After that, differentially expressed genes were aligned to the network by interacting with at least two seed genes. Subsequently, the clusters composed of genes closely interacting with each other were screened, and the genes in the clusters are thought to be more possibly to be pathogenic genes.

In the present work, the flow diagram of predicting pathogenic genes was shown in Figure 1. OMIM is a comprehensive and authoritative knowledge base of human genes as well as genetic disorders to support human genetics research and practice of clinical genetics [17]. Significantly, some identified pathogenic genes were deposited in OMIM database. Up to now, a total of 43 pathogenic genes of CC were deposited in OMIM database. In the current study, these 43 pathogenic genes were downloaded from the OMIM database for subsequent analysis. The intersection of these known pathogenic genes and the microarray data were extracted and called “seed genes.” Significantly, the seed gene list and the evidence that these genes were associated with CC were shown in Table 1 [18–38].

Human PPIN was obtained from the String database, and the seed genes and DEGs were aligned to the PPIN. Then, a new PPIN was extracted from the original PPIN, which was composed of seed genes and their adjacent DEGs. Additionally, a smaller subnetwork which was made up of genes interacting with at least two seed genes was detected from the new PPIN obtained above and identified as pathogenic network, where the genes in this pathogenic network were considered to be related to pathogenesis of CC.

Subsequently, in an attempt to identify the subnetworks in the pathogenic network, clustering with overlapping neighborhood expansion (ClusterONE), a plugin of Cytoscape [39], was utilized to carry out the cluster analysis.

### 2.4. Statistical Analysis of Prediction Results

In order to determine the significance of the predicted clusters, a significance score (SS) was defined for each cluster, where SS was considered as the geometric average of *P* values accompanying all the nodes in one cluster. The *P* value of each node was got via Wilcoxon test based on gene expression data of CC and control groups. In our background network, all the genes were differentially expressed, and the genes in one cluster were more differentially expressed when the genes were with smaller *P* values. In addition, if a set of genes were closely interacted and more differentially expressed, these genes were more likely referred to disease pathogenesis, since pathogenesis is generally involved in a set of genes which acted in concert. Hence, the SS herein was used to evaluate the significance of one cluster.

To determine the statistical significance of the predicted clusters, a *P* value was, respectively, computed for each cluster by means of randomization test. First of all, the *P* values of the genes in the cluster were randomly shuffled, and then each gene got a new *P* value after shuffling. Subsequently, we recalculated the SSs for the clusters after the *P* values were shuffled and these were identified as null distribution of SSs. Then, using the randomization test with 1,000 times, the *P* value for a cluster was determined as the probability that one cluster was identified in randomization test with smaller SS than that of our predicted cluster.

### 2.5. Identification of Candidate Genes

To select more accurate pathogenic genes from our method, each gene in the pathogenic network was assigned a weight value on the basis of the interactions as well as coexpressions with seed genes. If a gene interacted and was coexpressed with more seed genes, it was more likely to be a pathogenic gene. In detail, the coexpression between the predicted pathogenic gene and seed genes was calculated using Pearson correlation coefficients (PCCs) based on gene expression data. Then, the weight *w*(*x*) for each gene *x* was determined using PCCs. The weight of a gene was higher; the gene was more possible to participate in pathogenic procedure. Additionally, we determined the potential pathogenic genes as candidate genes of CC. The formula was listed as follows:

(1) $$\begin{array}{c} w\left(\;x\;\right)=\overset{}{\underset{y\in S}{\sum }}PC\left(\;x,y\;\right)I\left(\;x,y\;\right), \end{array}$$

where *S* was known pathogenic genes, PC(*x*, *y*) represented the PCC between gene *x* and gene *y*, and *I*(*x*, *y*) stood for an indication function; if protein *x* interacted with protein *y*, *I*(*x*, *y*) ~ 1; otherwise, *I*(*x*, *y*) ~ 0.

### 2.6. Pathway Enrichment Analysis of Candidate Genes and Seed Genes

Kyoto Encyclopedia of Genes and Genomes (KEGG) is a database that integrates genomic as well as systemic functional information, and KEGG offers a reference knowledge base for understanding cellular processes via the process of pathway aligning, which is to map genes to KEGG reference pathways to deduce systemic behaviors of the cell [40]. In our study, all the KEGG reference pathways were recruited from the KEGG database. Then, candidate genes and seed genes obtained in this work were aligned to these KEGG reference pathways to identify the potential pathways which were simultaneously enriched by candidate genes and seed genes. If candidate genes were involved in the same biological pathway with the seed genes, these candidate genes were potential pathogenic genes.

## 3. Results

### 3.1. Data Preprocessing and Identification of DEGs

Based on different platforms, a total of 5199 and 12329 genes were identified, respectively. Afterwards, 4654 overlapping genes in these two platforms were extracted. After data preprocessing; a total of 330 DEGs were identified under the delta value = 0.806.

### 3.2. Identification of Pathogenic Network

In the current study, a total of 43 known pathogenic genes of CC were downloaded from OMIM database and then mapped to the human PPIN. As a result, 21 of known pathogenic genes can be aligned to the PPIN, and these 21 genes were treated as seed genes (Table 1). Next, a subnetwork was extracted from the PPIN, and the genes in the subnetwork interacted with at least one seed gene.

**Figure 2:**
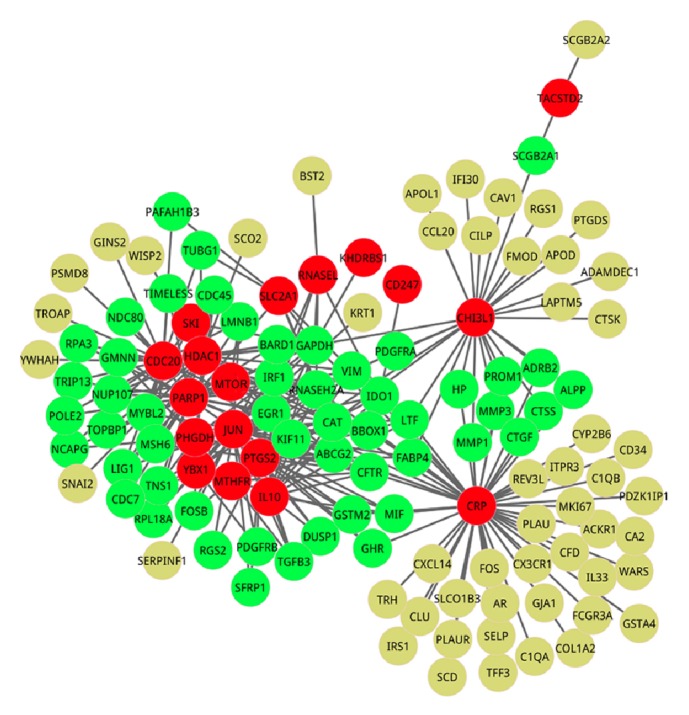
The new protein-protein interaction network (PPIN). The red nodes represent seed genes from OMIM database, that is, the known pathogenic genes, the green nodes denote genes interacting with at least two seed genes, and the yellow nodes are the genes interacting with only one seed gene. The new PPIN is composed of 123 nodes and 266 interactions. In addition, the genes in the new PPIN are differentially expressed in CC and control groups.

Although the genes interacting with seed genes might play important roles in maintaining the biological processes for CC development and progression, the usage of DEGs is helpful to reduce false positives since the expression changes of DEGs might be likely caused by the interactions with seed genes. After aligning DEGs to the original PPIN obtained above, we obtained a new PPIN which was composed of 123 genes and 266 interactions, as shown in Figure 2.

**Figure 3:**
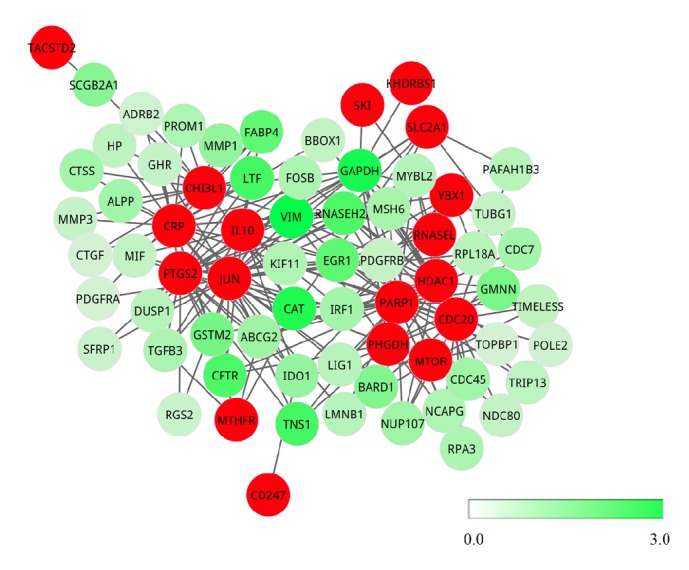
The pathogenic network. The red nodes represent seed genes from OMIM database, that is, the known pathogenic genes, the green nodes are genes interacting with at least two seed genes, and each node is assigned a weight value. The color bar stands for the relationship between color and weight: where the color is deeper, the weight is larger.

Subsequently, the genes interacting with at least two seed genes were screened out since these genes were more likely to be pathogenic genes. Herein, we identified the subnetwork which was made up of the genes interacting with at least two seed genes, and this subnetwork was named as pathogenic network hereafter, as exhibited in Figure 3. Importantly, we found that four seed genes, including* CRP*,* PTGS2*,* JUN*, and* IL-10*, interacted with each other and formed a clique. Hence, these four seed genes might belong to the same complex or pathway which is involved in the pathogenic process. In light of these results, the genes which interacted with these four seed genes tend more to be pathogenic genes. For instance,* LTF*, one iron-binding member of the transferrin family, has been demonstrated to regulate the tumor growth via mediating the transition from the G1 to S phase of cell cycle [41].

Moreover, a total of two intensely connected clusters were identified from the pathogenic network by employing Cytoscape. The genes in each cluster probably participated in the same signaling or regulatory pathway as seed genes, and these genes in cluster were more likely to be associated with pathogenic procedure.

**Figure 4:**
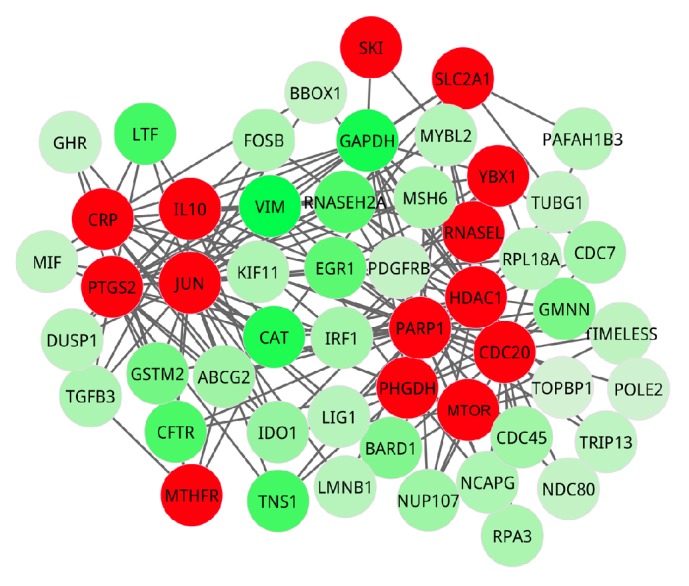
Cluster one. The red nodes represent seed genes from OMIM database, that is, the known pathogenic genes; the other nodes are genes interacting with seed genes. All the genes in the cluster were colored according to their weight values.

**Figure 5:**
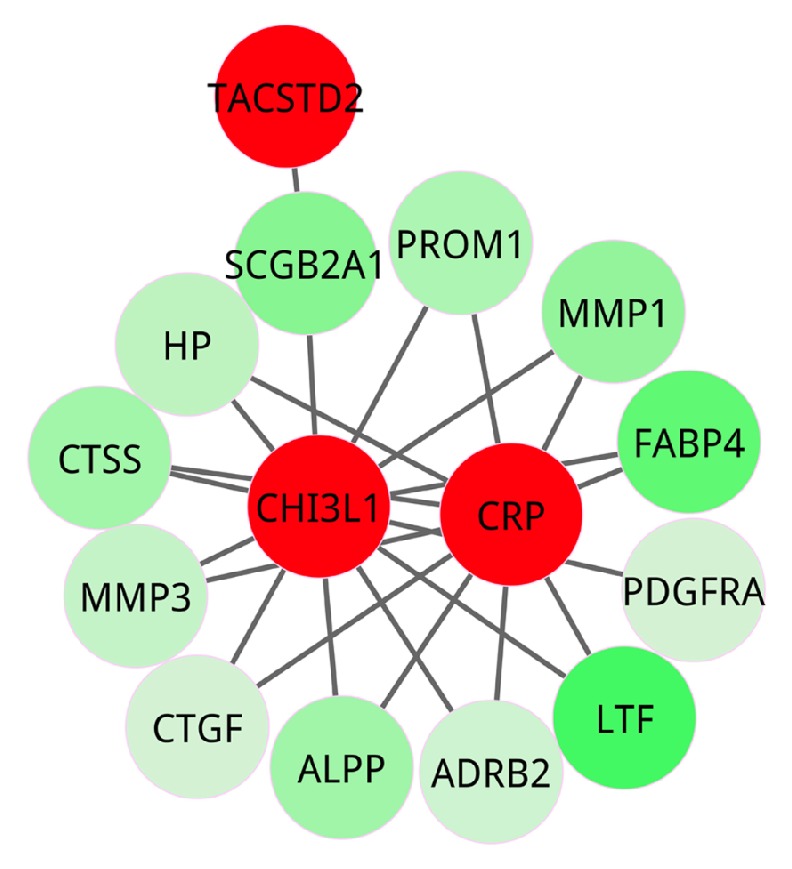
Cluster two. The red nodes are seed genes from OMIM database, that is, the known pathogenic genes; the other nodes stand for the genes interacting with seed genes. All the genes in the cluster were colored according to their weight values.

In cluster one, a total of 52 genes formed a tightly connected subnetwork, and 14 seed genes were contained as exhibited in Figure 4. In cluster two, there were 15 genes which formed a closely connected subnetwork involved in 3 seed genes (Figure 5).

### 3.3. Significance Analysis of Pathogenic Clusters

In an attempt to determine the importance of the clusters extracted above, the SS was defined for each cluster. Herein, the differential expression obtained *P* value was applied since a set of genes were more likely to be involved in pathogenesis if these were intensely connected in a network and more differentially expressed. It was noteworthy that a highly connected subnetwork did not imply that the genes in the subnetwork were remarkably differentially expressed. As a consequence, the SS score was utilized to determine whether a cluster can be identified by chance. In our study, the SS of cluster one and cluster two was 0.051 and 0.009, respectively.

To determine the significant difference of the two predicted clusters, a *P* value was derived for each cluster by means of the randomization test, respectively. The *P* values of the two clusters were, respectively, 1.4 × 10^−3^ and 5 × 10^−4^, which indicated that these two clusters were statistically significant and not identified by chance.

### 3.4. Identification of Candidate Genes

Based on the weight values of all the genes, we ranked these genes in descended order. Totally, 52 genes were identified when the weight values were more than 0.10. The top 20 genes with higher weight including* VIM*,* GAPDH*,* CAT*,* TNS1*,* LTF*,* CFTR*,* RNASEH2A*,* EGR1*,* FABP4*,* GSTM2*,* GMNN*,* BARD1*,* SCGB2A1*,* MMP1*,* IDO1*,* ABCG2*,* NUP107*,* CDC45*,* ALPP*, and* CTSS* were shown in Table 2.

### 3.5. Pathway Enrichment Analyses of Candidate Genes and Seed Genes

Pathway enrichment results showed that candidate genes and seed genes were simultaneously enriched in pathway in cancer, cell cycle, and methane metabolism (Table 3). Importantly,* MSH6*,* PTGS2*,* HDAC1*,* JUN*,* SLC2A1*, and* MMP1* were enriched in the pathway in cancer, of which* MSH6* and* MMP1* were candidate genes. Moreover, candidate genes* CDC7* and* CDC45*, as well as seed genes* HDAC1* and* CDC20,* were involved in the same pathway of cell cycle. Moreover, seed gene* MTHFR* and candidate gene* CAT* participated in the same pathway of methane metabolism.

## 4. Discussion

To illuminate the pathogenesis of CC, microarray profile E-GEOD-39001 was analyzed to predict pathogenic genes by means of a network method using known pathogenic genes as seed genes, where the genes with interaction of the known pathogenic genes were identified as candidate pathogenic genes due to the hypothesis that interacting proteins generally shared parallel functions. A total of 330 DEGs were identified in CC tissues. Moreover, two intensely connected clusters were extracted from the pathogenic network. Based on the weight values of all the genes in pathogenic network, 52 candidate genes were screened out when the weight values were more than 0.10. Among these,* VIM* had the highest weight value. Pathway results showed that seed genes* PTGS2*,* HDAC1*,* JUN*, and* SLC2A1* were enriched in pathway in cancer, seed genes* HDAC1* and* CDC20* were involved in cell cycle, and seed gene* MTHFR* participated in methane metabolism. Significantly, candidate genes* MMP1*,* CDC45*, and* CAT* were also, respectively, enriched in the pathway in cancer, cell cycle, and methane metabolism.

In literature, matrix metalloproteinases (MMPs), a group of proteases, have been indicated to play essential roles in the degradation of basement membrane as well as extracellular matrix (ECM) [42]. The deregulated ECM is demonstrated to result in the cell abnormal behaviors and the failure of organ function to lead to the incidence of cancer [43]. MMP1 is a member of MMPs, which exerts key functions in tumor invasion and metastasis in many cancers [44]. In particular, Nishioka and colleagues have revealed that* MMP1* promoter polymorphism influences the invasion of CC [45]. In our study,* MMP1* was found to be a candidate pathogenic gene and enriched in the pathway in cancer. Based on these results, we infer that* MMP1* in our predicted pathogenic network is tightly related with the pathogenic process and might be a pathogenic gene in the development of CC.

Cell cycle plays important roles in cell proliferation as well as cell growth [46]. It is well known that dysregulation of cell cycle brings about the aberrant cell proliferation which is a feature of a set of human cancers [47]. Significantly, Qin et al. [48] have indicated that CC cells are inhibited via inducing cell cycle arrest. In the current study, our findings argued for a significant role of cell cycle loss of control in the pathogenesis of CC, and a candidate pathogenic gene was screened out, for example,* CDC45*.* CDC45* exerts an important role in DNA replication including initiation and elongation phases [49]. A recent study has also indicated that* CDC45* has a key role in late G1 [50]. Defect in replication functions, for example,* CDC45*, leads to DNA damage as well as chromosome rearrangements [51]. This alteration of DNA replication can contribute to genome instability to further result in the development of cancer [52]. Thus, as demonstrated here,* CDC45* might be a promising pathogenic gene in biology process of CC.

Methane, as one of end products of fermentation in gastrointestinal system, rapidly appears in breath as do fermentation gases including hydrogen when methane is installed into the human colon [53]. Moreover, as early as 1992, Sivertsen et al. [54] have indicated that there is a relationship between breath methane and colorectal cancer, and the results are the same as that in 2013 offered by Holma et al. [55]. In our work, another potential pathogenic gene* CAT* was enriched in the pathway of methane metabolism.* CAT*, located on the chromosome 11 in human, has high GC content in the promoter [56]. Relative to normal samples,* CAT* has been indicated to be downregulated in tumor tissues [27, 57]. In particular, the decreased* CAT* is connected with the high concentration of hydrogen peroxide, which participates in the activation of pathways to lead to the proliferation, migration, and invasion of cancer cells [58]. Significantly, low level of* CAT* was observed in the CC patients [59]. Together, we infer that* CAT* is an important pathogenic gene to participate in the metabolism process to further cause the initiation of CC.

Interestingly, in the current study,* VIM* gene has the highest weight value in pathogenic network. As we all know, vimentin encoded by* VIM*, as a major member of the intermediate filament family, is especially expressed in connective tissue [60, 61]. It is noteworthy that vimentin exerts vital functions in cell adhesion, migration, and signaling [62]. Remarkably,* VIM* is an important target gene for various cancers. For example,* VIM* has been demonstrated to be methylated in advanced colorectal carcinomas and indicated to serve as a diagnostic biomarker in the detection and monitoring for colorectal carcinoma using stool and serum samples [63]. Moreover, Costa et al. [64] have suggested that* VIM*, as a marker, allows for early diagnosis of bladder cancer using urine samples. Jung et al. [65] also have suggested that* VIM* overexpression appears to positively influence the proliferation and migration in CC. In light of these, we infer that* VIM* might play important roles in the pathogenic process of CC.

## 5. Conclusion

In conclusion, our results provide evidence that candidate pathogenic genes such as* MMP1*,* CDC45*, and* CAT* and their enriched pathways, respectively, of pathway in cancer, cell cycle, and methane metabolism might be involved in the pathogenesis of CC. Moreover,* VIM* might play important roles in the pathogenic process of CC. We believe that the results obtained above can provide theoretical guidelines for future works in laboratory. Still, a mountain of work is warrant to understand the pathogenic process extensively.

## Figures and Tables

**Table 1:** The seed genes and the evidence that these genes are associated with cervical cancer.

Seed genes	Evidence
*MTHFR*	Mei et al., 2012 [18]
*MTOR*	Leisching et al., 2015 [19]
*PHGDH*	Jing et al., 2013 [20]
*CHI3L1*	Ngernyuang et al., 2014 [21]
*IL10*	Stanczuk et al., 2001 [22]
*CNKSR1*	Fritz and Radziwill, 2010 [23]
*BCL10*	Kuo et al., 2012 [24]
*PTGS2*	Ryu et al., 2000 [25]
*CD247*	Zehbe et al., 2006 [26]
*KHDRBS1*	Li et al., 2012 [27]
*SKI*	Chen et al., 2013 [28]
*CRP*	Polterauer et al., 2007 [29]
*TSPAN1*	Hölters et al., 2013 [30]
*HDAC1*	Lin et al., 2009 [31]
*YBX1*	Zhang et al., 2012 [32]
*RNASEL*	Madsen et al., 2008 [33]
*TACSTD2*	Varughese et al., 2011 [34]
*JUN*	Prusty and Das, 2005 [35]
*PARP1*	Hassumi-Fukasawa et al., 2012 [36]
*SLC2A1*	Airley et al., 2003 [37]
*CDC20*	Rajkumar et al., 2011 [38]

**Table 2:** The top 20 genes with higher weight values in pathogenic network.

Node	Weight value
*VIM*	2.622
*GAPDH*	2.491
*CAT*	2.386
*TNS1*	1.985
*LTF*	1.966
*CFTR*	1.886
*RNASEH2A*	1.880
*EGR1*	1.676
*FABP4*	1.646
*GSTM2*	1.354
*GMNN*	1.299
*BARD1*	1.190
*SCGB2A1*	1.118
*MMP1*	0.958
*IDO1*	0.931
*ABCG2*	0.829
*NUP107*	0.811
*CDC45*	0.795
*ALPP*	0.774
*CTSS*	0.761

**Table 3:** The KEGG pathway analysis of candidate genes and seed genes.

Terms	Candidate genes	Seed genes
Pathways in cancer	*MMP1*, *MSH6*	*PTGS2*, *HDAC1*, *JUN*, *SLC2A1*
Cell cycle	*CDC7*, *CD45*	*HDAC1, CDC20*
Methane metabolism	*CAT*	*MTHFR*
